# Meibomian Gland Dysfunction in Type 2 Diabetic Patients

**DOI:** 10.1155/2017/3047867

**Published:** 2017-05-16

**Authors:** Xiaolei Lin, Binbin Xu, Yuxi Zheng, Terry G. Coursey, Yinying Zhao, Junhua Li, Yana Fu, Xuewen Chen, Yun-e Zhao

**Affiliations:** ^1^School of Ophthalmology and Optometry, Eye Hospital, Wenzhou Medical University, Wenzhou, Zhejiang, China; ^2^Key Laboratory of Vision Science, Ministry of Health, Wenzhou, Zhejiang, China; ^3^Department of Ophthalmology and Visual Science, Eye, Ear, Nose, and Throat Hospital, Shanghai Medical College, Fudan University, Shanghai, China; ^4^Ocular Surface Center, Department of Ophthalmology, Cullen Eye Institute, Baylor College of Medicine, Houston, TX, USA

## Abstract

**Purpose:**

To investigate meibomian gland and tear film function in patients with type 2 diabetes.

**Methods:**

This prospective study compared changes in meibomian gland and tear film function in type 2 diabetic patients with nondiabetic patients. Meibomian gland function was evaluated by measuring lipid layer thickness (LLT), grading of meibomian gland loss, lid margin abnormalities, and expression of meibum. Tear film function was assessed by measuring tear breakup time (TBUT), the Schirmer I test, noninvasive breakup time (NIBUT), tear meniscus height (TMH), and corneal fluorescein staining.

**Results:**

Meibography scores were significantly higher in the diabetic group compared with the nondiabetic group (*p* = 0.004). The number of expressible glands was significantly lower in the diabetic group in temporal, central, and nasal third of the lower eyelid (nasal: *p* = 0.002; central: *p* = 0.040; and temporal: *p* = 0.039). The lid margin abnormality score was significantly higher in the diabetic group than in the nondiabetic group (*p* = 0.04). There was no statistically significant difference in the tear film function parameters between the two groups.

**Conclusions:**

Meibomian gland dysfunction (MGD) in type 2 diabetic patients is more severe compared with nondiabetic patients. Overall, most of the diabetic patients manifest as having asymptomatic MGD.

## 1. Introduction

Diabetes is a very common chronic disease and is a main cause of mortality and socioeconomic burden worldwide. The prevalence of type 2 diabetes has increased significantly in recent decades. According to a survey from US Department of Health and Human Services in 2001, there are nearly 25 million US people suffering from diabetes [1]. In a large epidemiological study of the Chinese adult population, the overall incidence of diabetes has reached 11.6% [2]. Ocular disorders are common in diabetic patients, such as retinopathy, corneal epithelial erosions, and dry eye [3, 4]. Despite the abundance of information available concerning the various complications of chronic diabetes, the effects of this disease on ocular disorders, particularly dry eye, are not yet fully appreciated. The dry eye workshop (DEWS) of 2007 indicated that diabetes may be a risk factor of dry eye [5]. A large proportion of diabetic patients suffer from dry eye that results in visual disturbance and often interfere with their quality of life. Tear function in diabetic patients, especially patients suffering proliferative diabetic retinopathy, was commonly worse than nondiabetic people [6]. The outcome of another large epidemiologic study in Spain suggested that diabetes was associated with asymptomatic meibomian gland dysfunction (MGD), a major cause of ocular discomfort and inflammation [7].

The meibomian gland synthesizes and produces lipids and proteins which form the outermost layer of the tear film. These lipids decrease evaporation and promote stability of the tear film. The International Workshop on Meibomian Gland Dysfunction suggests that MGD is the most prevalent cause of evaporative dry eye and may play a role in aqueous-deficient dry eye [8]. Recently, a study from Ding et al. demonstrated that insulin stimulated the proliferation of immortalized human meibomian gland epithelial cells (HMGECs), whereas high glucose was found to be toxic for HMGECs [9]. This suggests that insulin resistance/deficiency and hyperglycemia are deleterious for HMGECs which supports our hypothesis that diabetes may be associated with MGD. However, there have been few clinical studies examining meibomian gland function in type 2 diabetes. To address this, we conducted a prospective randomized controlled trial that aimed to investigate meibomian gland and tear film function in type 2 diabetic patients.

## 2. Materials and Methods

### 2.1. Subjects

The study was approved by the Investigational Review Board of School of Ophthalmology and Optometry and Eye Hospital, Wenzhou Medical University, Wenzhou, China. All subjects enrolled gave informed consent prior to their inclusion in the study. Patients who had been previously diagnosed with type 2 diabetes by a physician were enrolled in the study group. For the control group, fasting blood glucose was measured to rule out diabetes even without a history of diabetes. Thirty-nine type 2 diabetic patients and fifty-four nondiabetic patients were recruited into the study between May 31, 2015, and December 28, 2015. Both eyes of each patient were evaluated. There was no significant difference in age and gender between the two study groups. The inclusion criteria were as follows: at least 40 years of age and willingness to participate in the study. The exclusion criteria were as follows: active ocular infection or inflammation, previous ocular surgery, continuous use of topical ocular medications, and a history of cranial nerve injury or any other diseases known to affect the tear film.

Each patient completed an ocular surface disease index (OSDI) questionnaire for the assessment of ocular surface symptoms. Subjects were considered symptomatic if the value was 12 or greater [10]. All patients underwent a series of ocular surface examinations in the following order: lipid layer thickness (LLT), tear meniscus height (TMH), noninvasive breakup time (NIBUT), bulbar and limbal hyperaemia, corneal fluorescein staining, tear breakup time (TBUT), the Schirmer I test, grading of meibomian gland loss, and expression of meibum. An interval of at least 10 minutes between every two examinations was assured. All the patients were examined by the same physicians (L. X. L. and C. X. W.).

### 2.2. Evaluation of Meibomian Gland Function

The morphology of meibomian glands was evaluated by Keratograph 5M (Oculus GmbH, Wetzlar, Germany). The upper and lower eyelids were ectropionized and images were captured. We obtained the meibography scores, which quantitate the dystrophy and obstruction of the meibomian glands, using the grading system of Arita et al. The sum of the upper and lower lids scores was recorded as the total meibography score ranging from 0 to 6 [11].

The number of expressible meibomian glands was quantified using the meibomian gland evaluator (TearScience Inc., Morrisville, NC). A stable pressure was applied to the temporal, the central, and the nasal third of the lower eyelid, and the number of secretable glands was recorded [12].

Lid margin abnormalities were scored according to the following 4 signs: vascular engorgement, lid margin irregularity, obstructed meibomian gland orifices, and anterior or posterior displacement of the mucocutaneous junction [13]. The lid margin score was from 0 to 4.

### 2.3. Tear Film Measurement Using the Keratograph 5M

Tear films of all the subjects were scanned by the corneal topographer Keratograph 5M to assess the quality of the tear film. Subjects were seated at the Keratograph 5M in a darkened room with the subject eye focused on the central target that consists of a placido disc comprised of 22 illuminated rings, which projected onto the corneal surface. The lower tear film meniscus images were captured 5 s after blinking, and the values were obtained by the caliper function of the software. Using the technology, TMH, centered on the inferior cornea and lower eyelid, was measured. This was repeated three times.

For NIBUT measurement, the subjects were instructed to blink two times and then keep their eyes open to the best of their ability. The first NIBUT (NIBUT-1st) and the average TBUT (NIBUT-avg) (average value of TBUT across the observed area) were calculated automatically by the software. The test was repeated two times for each eye, and the average was recorded.

For bulbar and limbal hyperaemia assessment, patients were required to open his or her eyes as large as possible and focus on a point inside the camera while a keratograph image was captured. The software analyzes the image automatically and assigns a red eye index (accurate to 0.1 unit).

### 2.4. Lipid Layer Thickness (LLT)

LLT was determined using the LipiView interferometer (TearScience Inc.). The system software analyzes the interference pattern of the tear film and assigns a value in interferometric color units (ICUs), which reflects the thickness of the tear film.

### 2.5. Tear Film Breakup Time (TBUT)

TBUT was calculated after placing a fluorescein strip into the lower conjunctival fornix. The interval between the very last complete blink and the very first break spot was recorded. The average value of three measurements was recorded.

### 2.6. Corneal Fluorescein Staining (FL)

The fluorescein instillation method was the same as TBUT. The intensity of corneal fluorescein staining was graded using the Baylor grading scheme [14].

### 2.7. The Schirmer I Test (Schirmer)

The Schirmer I tests were performed without anesthetics. A sterile strip was inserted in the midlateral portion of the inferior fornix, and the patient was instructed to close their eyes for five minutes. The lengths of wet area of strips were measured and recorded.

### 2.8. Statistical Analysis

The data were analyzed using SPSS 20.0 (SPSS, Chicago, IL). Results of the descriptive statistics are presented as the mean ± standard deviation or median (interquartile range (IQR)). All data sets were tested for normality using the Kolmogorov-Smirnov test. For data that were normally distributed, the independent-samples *t*-test was employed to compare the results of the diabetic group and nondiabetic subjects. The correlations between the duration of diabetes, meibomian gland function, and tear film variables in the diabetic group were studied using Pearson's correlation coefficient. For data that were not normally distributed, the Mann–Whitney test was used to compare the results of the two groups. The correlations between the duration of diabetes, meibomian gland function, and tear film variables were studied using Spearman's correlation coefficient. The chi-square test was used to compare gender ratios between groups. *p* values less than 0.05 were considered statistically significant.

## 3. Results

### 3.1. Description of Enrolled Subjects

From May 2015 to December 2015, we prospectively enrolled 39 type 2 diabetic patients (23 women and 16 men; age 67.05 ± 1.53 years, range 40–87) and 54 nondiabetic patients (31 women and 23 men; age 67.19 ± 1.72 years, range 41–83). In the diabetic group, the average duration of diabetes was 9.06 ± 5.39 years. And the mean fasting plasma glucose was 7.75 ± 1.43 mg/dl. 52.8% of the participants used oral glucose-lowering drugs (OGLDs), while 33.3% took insulin, and 13.9% were on OGLDs plus insulin. In the nondiabetic group, the fasting plasma glucose was 5.80 ± 1.26 mg/dl.  Table 1 summarizes the demographic and clinical characteristics of the participants. The age and gender did not differ significantly between diabetic and nondiabetic groups. The fasting plasma glucose in the diabetic group was significantly higher compared to the nondiabetic group (*p* < 0.001).

### 3.2. Meibomian Gland Morphology and Dysfunction in Diabetic Patients Compared with Nondiabetic Patients

The meibography score was significantly higher in the diabetic group compared with the nondiabetic group (*p* = 0.004). In the diabetic group, the mean value of the meiboscore was 4.25 ± 1.35. By contrast, in the nondiabetic group, the mean value was 3.62 ± 1.41.

Additionally, the number of expressible glands was significantly different between the two groups at all of the temporal, central, and nasal third of the lower eyelid (nasal: *p* = 0.002; central: *p* = 0.040; and temporal: *p* = 0.039). The lid margin abnormality score was also significantly higher in the diabetic group compared to the nondiabetic group (*p* = 0.04). Lipid layer thickness was significantly higher in the diabetic group (*p* = 0.019). These results are found in Table 2.

### 3.3. Comparison in Tear Film Parameters between Diabetic and Nondiabetic Groups

No significant differences were observed in TBUT, the Schirmer I test, TMH, NIBUT, bulbar and limbal hyperaemia, and corneal fluorescein staining between the diabetic and nondiabetic groups. Patient information and results of tear parameters are found in Table 1. These results suggest that diabetic patients suffer more severe meibomian gland dysfunction than the nondiabetic patients, although aqueous tear production was in normal ranges and no difference was observed between these two groups.

### 3.4. Comparison in Meibomian Gland Function and Tear Film Indexes between Symptomatic and Asymptomatic Patients within the Diabetic Group

In the diabetic group, 41.03% (16/39) patients were symptomatic and 58.97% (23/39) were asymptomatic. Among the diabetic patients, 30.77% (12/39) required dry eye therapy. TBUT was significantly higher in asymptomatic patients (*p* < 0.001). However, no significant difference was observed in meibomian gland function and other tear film indexes between symptomatic and asymptomatic patients. The results are found in Table 3.

### 3.5. Correlations of Meibomian Gland Dysfunction Parameters with Dry Eye Indexes in Diabetic Patients

Spearman correlation analysis showed that the history of diabetes is significantly correlated with TBUT (*R* = −0.472, 95% confidence interval: −0.644 to −0.299, *p* < 0.001). TBUT is also inversely correlated with limbal hyperaemia (*R* = −0.341, *p* = 0.024), TMH (*R* = −0.239, *p* = 0.04), and OSDI (*R* = −0.350, *p* = 0.034). There was no significant correlation between other measured parameters.

## 4. Discussion

Our study revealed that meibomian gland morphology and dysfunction, including meibography scores, lid margin abnormalities, and meibum expressibility, were found to be significantly worse in patients with type 2 diabetes. However, no change in the OSDI, the Schirmer I test, TBUT, NIBUT, TMH, or corneal fluorescein staining was observed. These results suggest that type 2 diabetic patients may be at a greater risk of developing meibomian gland dysfunction.

Although our study provides evidence that the meibomian gland function was significantly impaired in diabetic patients, the mechanism of this is unknown and warrants further research. The World Health Organization Global Burden of Disease Study reported that 220 million people suffer from type 2 diabetes mellitus (DM) in 2010 and by the year 2030, a rise to 366 million is predicted [15, 16]. Long-term duration of the disease and insufficient control of blood glucose result in the prevalence of various complications, among which, peripheral neuropathy belongs to the first few ones [17].

During the course of peripheral neuropathy, A*δ* and unmyelinated C-class small nerve fibres were gradually damaged [18]. Damage to the corneal nerves has both morphological and functional consequences. Changes in the morphology of corneal nerves cause the reduction in subbasal nerve density, decreased nerve branching, and increased nerve tortuosity [19–21]. Functionally, damage to the corneal nerves results in decreased corneal sensitivity, a fact well-documented in the literature [22–25]. Decreased corneal sensitivity, which reduces the blink rate, leads to the destabilization of the lipid layer of the tear film and results in increased excess evaporation [26]. Peripheral neuropathy may also be associated with MGD, as the contraction of lid muscles facilitates the delivery of the lipid from the meibomian gland. During the blink movement of the eyelids, the orbicularis muscle produces a compression force to the tarsal plate and the enclosed tarsal glands of meibom [27]. The contraction of Riolan's muscle exerts pressure to the end part of the ductal system and acini and is beneficial to the delivery of the lipid to the lid margin [27]. Decreased corneal sensitivity may influence the control of orbicularis and Riolan's muscle in DM patients and may be a reason for increased MGD in DM patients.

Furthermore, the concomitant inflammatory response with diabetes may also induce MGD. Suzuki et al. have suggested that obstructive MGD is a precursor of meibomitis [28]. Meibomitis, an inflammatory form of MGD, has been associated with the ocular surface inflammation. The proposed mechanism is that inflamed meibomian gland orifices in meibomitis release a pathogen-related substance onto the ocular surface and results in cell-mediated ocular surface inflammation [28]. Baudouin et al. further suggest that pathological mechanisms of MGD may be the result of stasis of the meibum, promoting the growth of bacteria and increasing the release of esterases and lipases [29]. A consequence of increased enzymatic activity and bacterial growth is an increase in the viscosity of the meibum and generation of free fatty acids (FFAs), which in turn induces inflammation and hyperkeratinisation [27, 29]. In diabetes, it has been recognized that plasma FFAs can cause insulin resistance [30] and increase the expression and release of proinflammatory cytokines [31]. This leads us to hypothesize that increased FFAs in the meibomian glands may lead to the accumulation of the meibum resulting in increased inflammation that leads to meibomitis. Further studies must be conducted to test this hypothesis and is the subject of ongoing work.

Additionally, insulin is essential for the desired sebaceous gland activity and is known to induce glandular cell proliferation and lipid secretion. Second, hyperglycemia has been shown to contribute to lipolysis in adipocytes. Ding et al. reported that hyperglycemia could lead to morphologic changes and a gradual loss of HMGECs, which indicates that in diabetic patients, hyperglycemia is one of the pathogenic factors causing MGD [9]. This study provides a putative molecular mechanism to explain the correlation of diabetes with human MGD. MGD is the most common cause of evaporative dry eye and is associated, to some degree, with aqueous-deficient dry eye.

In another study, Shamsheer and Arunachalam also found that diabetes is associated with MGD [32]. However, this study only measured the MG expression scale, symptoms, and corneal staining. It did not measure the morphology of meibomian gland, the lid margin abnormalities, and other tear film parameters. To the best of our knowledge, the present investigation is the first study that focuses on meibomian gland atrophy and meibomian gland expressibility in diabetic patients and provides additional evidence for the correlation between MGD and DM.

Interestingly, our results indicate that the LLT was thicker in the diabetic group. This seems counterintuitive and deserves further investigation. Finis et al. reported a decrease in variation of the LLT during the day. Measurements also differed day to day compared to the tear film breakup time [33]. It is possible that the fluctuation of LLT during the course of the day may explain these results.

However, our study found no significant differences in tear film parameters between the diabetic and nondiabetic groups. In previously published reports, the conclusions regarding TBUT, Schirmer, or other parameters in patients with DM are controversial. Some studies suggested no difference [34–36], and others demonstrated a decreased TBUT and Schirmer [37, 38]. Our study demonstrated a nonstatistically significant increase in dry eye signs. One reason for this difference may be that patients who participated in this study were also scheduled for cataract surgery, and most of them maintain stable levels of blood glucose. Furthermore, the mean duration of diabetes in our study was relatively shorter. Several studies reported that dry eye in diabetes was related to the duration of diabetes [6, 37]. In addition, a few studies found that tear function was associated with PDR and poor metabolic glucose control [39, 40]. Therefore, the selection bias in the inclusion of diabetic patients may influence these results.

Furthermore, our study analyzed the correlations among meibomian gland abnormalities, subjective symptoms, and tear film parameters. We found that in the diabetic group, 41.03% had dry eye symptoms and 30.77% required dry eye therapy. However, there was no significant differences between symptomatic and asymptomatic patients in meibomian gland function and tear film parameters. Only TMH showed a negative correlation with OSDI. No significant correlation was found with the other measured parameters. These findings suggest that diabetic patients may suffer with ocular surface disorders even if dry eye symptoms are absent. Viso et al. also reported that diabetes was associated with asymptomatic MGD [7]. The absence of symptoms among diabetic patients may be due to the impairment of sensory peripheral nerves. Besides, these results suggest that symptoms in patients with diabetes may not be associated with signs of MGD and dry eye. Similar findings have been reported with other ocular surface disorders [41, 42]. However, further investigation is needed to confirm these results.

In summary, our data suggest that MGD in diabetic patients is more severe compared with nondiabetic patients. Limitations of our study include small sample size, and we did not account for differing severities of diabetes in our patients. Further studies are needed to expand the sample size and compare meibomian gland function between nonproliferative diabetic retinopathy, proliferative diabetic retinopathy, and nondiabetic retinopathy patients. The understanding of the role of FFAs in the development of MGD in diabetic patients requires future studies.

## Figures and Tables

**Table 1:** Patient information and results of tear parameters in the diabetic group and nondiabetic group.

Parameters	Diabetic group *n* = 39 (78 eyes)	Nondiabetic group *n* = 54 (108 eyes)	*p*
Age(yr)	67.05 ± 1.53	67.19 ± 1.72	0.338
Sex ratio (male/female)	16/23	23/31	0.306
Fasting blood glucose (mg/dl)	7.75 ± 1.43	5.80 ± 1.26	<0.001
OSDI	12.79 ± 13.91	13.55 ± 16.42	0.975
TBUT (s)	3.79 ± 2.25	3.99 ± 2.60	0.487
Fluorescein score	1.77 ± 2.49	1.40 ± 2.72	0.076
Schirmer (mm)	5.57 ± 4.70	6.55 ± 5.93	0.431
TMH (mm)	0.19 ± 0.10	0.22 ± 0.14	0.131
NIBUT-1st (s)	6.46 ± 4.13	6.92 ± 4.70	0.849
NIBUT-avg (s)	8.59 ± 4.94	9.53 ± 5.61	0.448
Bulbar hyperaemia	1.47 ± 0.51	2.04 ± 0.54	0.210
Limbal hyperaemia	1.90 ± 0.55	1.61 ± 0.52	0.167

**Table 2:** Comparison of meibomian gland parameters in the diabetic group and nondiabetic group.

Parameters	Diabetic group	Nondiabetic group	*p*
LLT	77.21 ± 21.24	67.52 ± 24.10	0.019
Meibography score	4.25 ± 1.35	3.62 ± 1.41	0.004
Lid margin abnormality score	2.47 ± 0.75	2.26 ± 0.75	0.04
The number of expressible glands			
Temporal third	0.62 ± 1.02	1.88 ± 1.70	0.039
Central third	1.05 ± 1.59	1.62 ± 1.59	0.040
Nasal third	0.82 ± 1.10	1.88 ± 1.90	0.002

**Table 3:** Comparison of meibomian gland function and tear film parameters between the symptomatic and asymptomatic patients in the diabetic group.

Parameters	Symptomatic patients	Asymptomatic patients	*p*
The history of diabetes	12.69 ± 8.32	9.73 ± 5.99	0.305
Fasting blood glucose (mg/dl)	8.71 ± 4.47	9.74 ± 5.89	0.884
OSDI	25.41 ± 13.45	4.01 ± 3.85	<0.001
TBUT (s)	2.71 ± 0.88	4.62 ± 2.60	<0.001
Fluorescein score	1.75 ± 2.24	1.79 ± 2.70	0.745
Schirmer (mm)	5.12 ± 3.47	5.30 ± 3.90	0.932
TMH (mm)	0.18 ± 0.10	0.19 ± 0.10	0.729
NIBUT-1st (s)	6.46 ± 5.34	7.63 ± 5.83	0.311
NIBUT-avg (s)	8.22 ± 6.37	9.86 ± 6.61	0.198
LLT	79.40 ± 22.32	75.16 ± 20.31	0.289
Meibography score	3.90 ± 1.40	4.54 ± 1.26	0.067
Lid margin abnormality score	2.50 ± 0.66	2.44 ± 0.81	0.892
The number of expressible glands			
Temporal third	1.42 ± 1.88	1.26 ± 1.58	0.948
Central third	1.00 ± 1.60	1.56 ± 1.50	0.153
Nasal third	0.75 ± 1.36	1.00 ± 1.24	0.271
